# Prevalence of anticipatory nausea and vomiting in breast cancer patients undergoing highly emetogenic chemotherapy

**DOI:** 10.1590/1806-9282.20230937

**Published:** 2024-05-03

**Authors:** Rafaela de Brito Alves, Camilla Vieira de Rebouças, Alayne Magalhães Trindade Domingues Yamada, Felipe José Silva Melo Cruz

**Affiliations:** 1 Faculty of Medicine of ABC, Brazilian Institute for Cancer Control – Santo André (SP), Brazil.; 2 Brazilian Institute for Cancer Control – São Paulo (SP), Brazil.

**Keywords:** Nausea, Vomiting, Antineoplastic protocols

## Abstract

**OBJECTIVE:**

Anticipatory nausea and vomiting are unpleasant symptoms observed before undergoing chemotherapy sessions. Less is known about the occurrence of symptoms since the advent of the new neurokinin-1 antagonist.

**METHODS:**

This prospective cohort study was performed at a single Brazilian Institution. This study included breast cancer patients who received doxorubicin and cyclophosphamide chemotherapy and an appropriate antiemetic regimen (dexamethasone 10 mg, palonosetron 0.56 mg, and netupitant 300 mg in the D1 followed by dexamethasone 10 mg 12/12 h in D2 and D4). Patients used a diary to record nausea, vomiting, and use of rescue medication in the first two cycles of treatment. The prevalence of anticipatory nausea and vomiting was assessed before chemotherapy on day 1 of C2.

**RESULTS:**

From August 4, 2020, to August 12, 2021, 60 patients were screened, and 52 patients were enrolled. The mean age was 50.8 (28–69) years, most had stage III (53.8%), and most received chemotherapy with curative intent (94%). During the first cycle, the frequency of overall nausea and vomiting was 67.31%, and that of severe nausea and vomiting (defined as grade>4 on a 10-point visual scale or use of rescue medication) was 55.77%. Ten patients had anticipatory nausea and vomiting (19.23%). The occurrence of nausea and vomiting during C1 was the only statistically significant predictor of anticipatory nausea and vomiting (OR=16, 95%CI 2.4–670.9, p=0.0003).

**CONCLUSION:**

The prevalence of anticipatory nausea is still high in the era of neurokinin-1 antagonists, and failure of antiemetic control in C1 remains the main risk factor. All efforts should be made to control chemotherapy-induced nausea or nausea and vomiting on C1 to avoid anticipatory nausea.

## INTRODUCTION

Chemotherapy-induced nausea and vomiting (CINV) are common treatment-related side effects that worsen the quality of life and adherence and may lead to dose reductions or discontinuation^1,2^. Approximately 70–80% of patients receiving chemotherapy are at risk of developing nausea and vomiting^3^. In the past, they were unavoidable side effects, leading patients to postpone or refuse potentially curative treatments. Since the late 1980s, drugs such as dopamine and 5-HT3 receptor antagonists, and later neurokinin-1 antagonists, have enabled greater control of CINV^1,2,4,5^.

The main risk factor for CINV is the emetogenic potential of the chemotherapeutic agent. Chemotherapy regimens are classified as having high (>90% chance of nausea and vomiting), moderate (30–90%), low (10–30%), and minimal emetogenic potential (<10%)^2,4,6^. Other risk factors related to CINV are young age, female sex, history of nausea and vomiting during pregnancy, and vomiting in previous chemotherapy, while alcohol consumption is a protective factor^1,2,7^. Multiple mechanisms are involved in the appearance of CINV^8^, which differ according to when the condition manifests: acute, late, and anticipatory. The acute period is the first 24 h of antineoplastic drug administration, while the late period begins after 24 h after chemotherapy administration, usually 2–3 days after infusion. Anticipatory nausea and/or vomiting occurs when an adverse memory triggers nausea and/or vomiting before chemotherapy administration^1,2^.

Anticipatory nausea and vomiting (ANV), also referred to as conditioned (learned) nausea and vomiting to chemotherapy, are described in approximately 25% of chemotherapy patients. The risk tends to increase with the number of cycles received and may persist after the end of chemotherapy. Most studies on ANV were performed before the introduction of neurokinin-1 inhibitors.

The primary objective of this study was to evaluate the prevalence of ANV in cancer patients undergoing highly emetogenic chemotherapy who received adequate antiemetic prophylaxis, including corticosteroids, 5-HT3 antagonists, and neurokinin-1 inhibitors. The secondary objective was to find predictors of ANV.

## METHODS

A prospective cohort study was conducted in an oncology center located in the city of São Paulo, where patients were recruited from August 2020 to August 2021. Eligible patients were adults (≥18 years old) with breast cancer who had never received highly emetogenic chemotherapy and were scheduled to receive at least two cycles. We excluded patients who were unable to complete the diary or made incorrect use of emetic prophylaxis, patients who reported symptoms of nausea or vomiting before the first cycle of treatment, and patients who presented with a pathology or condition that caused emesis (central nervous system metastasis, gastrointestinal obstruction, metabolic or electrolyte disorders, alcohol abuse, or opioid use).

For data collection, a questionnaire prepared by the researchers was used with information containing sociodemographic characteristics, clinical data, and data referring to chemotherapy treatment, in addition to the regimen used to prevent nausea and vomiting. The occurrence of nausea and vomiting at home was assessed using a diary in which the patients recorded each episode and the use of rescue antiemetic medications, in addition to grading the intensity of symptoms according to the visual analog scale (VAS), to be completed after each cycle of chemotherapy, for two consecutive cycles. Immediately before the second cycle of chemotherapy, patients were evaluated for the occurrence of ANV, defined as the occurrence of nausea and/or vomiting up to 24 h before the infusion of chemotherapy.

The study was conducted in accordance with the ethical principles of international guidelines such as the Declaration of Helsinki and the ICH-GCPC Guideline and was approved by the research ethics committee of the institution (Opinion No. 4128120/ClinicalTrials.gov number, NCT04785495). All patients signed an informed consent form.

Categorical variables were described according to frequency distribution, and continuous variables were described with summary measures (mean, standard deviation, median, minimum, and maximum). Fisher's test was used to evaluate the association of clinical characteristics with the occurrence of ANV. The McNemar test was used to evaluate the association between nausea and vomiting in the first cycle and the occurrence of ANV. The analyses were performed using the Stata 17 software, and a significance level of 5% was considered.

To calculate the sample size needed, we estimated that the prevalence of ANV would be 20%. We estimated that the inclusion of 50 patients in the triage phase (first cycle) would lead to the occurrence of nausea and vomiting in the second cycle in 10 patients, which would allow us to run univariate analyses to find predictive factors of ANV. Assuming a 20% loss to follow-up, we screened 60 patients.

## RESULTS

Between August 2020 and August 2021, 60 patients were recruited, of whom 52 were considered eligible and were included in the study. All patients received the same chemotherapy regimen, doxorubicin (60 mg/m²) and cyclophosphamide (600 mg/m²). The regimen used to prevent nausea and vomiting was composed of dexamethasone 10 mg, palonosetron 0.56 mg, and netupitant 300 mg in the D1 followed by dexamethasone 10 mg 12/12 h in D2 and D4.

The sociodemographic and clinical characteristics of the patients are shown in Table 1. The median age was 50.2 years (interval 28–69), and all patients were female. Regarding the risk factors for nausea and vomiting, 43.48% reported a history of nausea or vomiting during pregnancy.

**Table 1:** Sociodemographic and clinical characteristics.

	No.	%		No.	%
**Gender**			**Age (years)**		
Woman	60	100	Median	50.2	–
			Range	28–69	
**Number of children**			**Marital status**		
None	6	11.54	Single	11	21.15
At least 1	46	88.46	Married/stable union	28	53.85
			Separated/widowed	13	25
**Molecular characteristics**			**Staging**		
RE positive	22	42.31	I	1	1.92
RE negative	30	57.69	II	20	38.46
HER 2 positive	38	73.08	III	28	53.85
HER 2 negative	14	26.92	IV	3	5.77
**Treatment**			**Chemotherapy regime**		
Neoadjuvant/adjuvant	49	94.23	AC*	31	59.62
Palliative	3	5.77	Dense dose AC3**	21	40.38

RE: estrogen receptor; HER2: human epidermal growth factor receptor type 2; RE: estrogen receptor; HER2: human epidermal growth factor receptor type 2;

* doxorubicin (60 mg/m²) and cyclophosphamide (600 mg/m²), every 3 weeks;

** doxorubicin (60 mg/m²) and cyclophosphamide (600 mg/m²), every 2 weeks.

Acute nausea and vomiting (in the first 24 h after infusion) in the first cycle were reported by 30 patients (57.69%), with rescue medication use in 40.38% of the sample. Delayed nausea and vomiting, after 24 h, were recorded in 57.69% of patients, with the need for rescue agents in 42.31% of patients. Overall, 35 patients (67.31%) reported acute or delayed nausea and vomiting, and 55.77% of the patients rated the symptoms as ≥4 (moderate) or took a rescue drug (Table 2).

**Table 2:** Occurrence of acute and delayed nausea and vomiting in the first cycle.

		No.	%
**Acute**			
	Yes	30	57.69
	No	22	42.31
**Use of rescue drug**			
	Yes	21	40.38
	No	31	59.62
**Late**			
	Yes	30	57.69
	No	22	42.31
**Use of rescued rug**			
	Yes	22	42.31
	No	30	57.69
**Acute or late**			
	Yes	35	67.31
	No	17	32.69
≥**4 or rescue use**			
	Yes	29	55.77
	No	23	44.23

The prevalence of ANV was 19.23% (n=10). The occurrence of nausea and vomiting during the first cycle was the only factor statistically associated with the onset of ANV symptoms (OR=16, 95%CI 2.4–670.9, p=0.0003; Table 3). We did not observe an association between age, history of nausea and vomiting in previous pregnancies, treatment intention, or regimen, and the occurrence of ANV.

**Table 3:** Relationship between the occurrence of nausea/vomiting in C1 and anticipatory nausea/vomiting before C2.

	Pre-C2 anticipatory nausea/vomiting		OR	p-value
**Nausea/vomiting in C1**	**Yes**	**No**		
Yes	9	16	16	0.0003
No	1	26	95%CI 2.4–670.9	

## DISCUSSION

The objective of this study was to measure the prevalence of ANV in patients undergoing highly emetogenic chemotherapy and who used optimal prophylaxis. We also sought the factors associated with the onset of symptoms.

Our results suggest that ANV remains a prevalent problem, as it was reported by approximately 20% of the patients in the study. The data presented indicate that despite antiemetic prophylaxis with 5-HT3 antagonists and NK1 inhibitors, considered the gold standard by international protocols^4,9,10^, the overall management of these events remains a challenge that deserves attention. The prevalence of ANV found in the study was slightly higher than that reported in some studies^9,11^. Overall, women and young age are both risk factors for this symptomology^1,2,6^. However, such variations in the prevalence of ANV have already been found in previous studies, which is explained by the differences between the populations and the chemotherapy regimens evaluated in each study^9-12^.

In this study, nausea and vomiting in the first cycle were the only significant predictors of ANV before the second cycle (p-value 0.0003). Thus, the control of nausea and vomiting from the first cycle is essential to reduce the prevalence of ANV before the second cycle and possibly in later cycles.

There is a well-established relationship between the noncontrol of CINV in early cycles and the onset of anticipatory symptoms in later cycles. This relationship is explained by the conditioning component and is particularly linked to psychological processes or previous experiences with the symptoms of ANV^13-15^. The use of adjuvant therapies based on behavioral or psychological interventions, such as music therapy, mindfulness, acupuncture, inhaled aromatherapy, and hypnosis, can help control anticipatory symptoms^9,10,16-19^, which reinforces the role of psychological processes in the emergence of these symptoms. It is believed that drug therapy, then, may occupy an adjuvant position and that when it is used in combination with behavioral therapies for the management of CIVN, we may achieve higher rates of control of ANV.

Historically, nausea and vomiting have been studied concomitantly considering the same physiological mechanisms. However, vomiting, when compared with nausea, has been better controlled and the evolution of new therapies suggests that mechanisms for the development of symptoms are different^4^.

Other epidemiological and clinical variables were not associated with ANV here. A history of nausea and vomiting during pregnancy was associated as a risk factor for the onset of ANV in previous studies. It could be that the improvement of antiemetic therapy, including for symptom control during pregnancy may explain this finding in our study. Intention-to-treat approach, education level, and race are no longer considered predictive factors of symptoms, and indeed, we observed no relationship between these factors and the onset of ANV.

Another relevant aspect identified during this study is the way in which patients who initiated treatment understood the information we gave them. Professionals and cancer centers should strengthen surveillance for the identification of potential flaws that contribute to the emergence of CINV. For example, lack of knowledge of rescue antiemetic therapy, lack of access to drugs, and inappropriate use of therapies are relatively simple improvement points for controlling such symptoms.

Guideline recommendations for the management of anticipatory NV focus on its prevention through the use of optimal antiemetic therapy for each cycle of chemotherapy. Therefore, it is concluded that nausea and vomiting are more easily prevented than treated. One of the limitations of this study was the small number of patients analyzed, all of whom took the same chemotherapy regimen. We also considered a limiting factor the fact that the symptoms were analyzed only before cycle 2 and were not studied in later cycles, which may have led us to underestimate the occurrence of nausea and vomiting in our population.

## CONCLUSION

The prevalence of ANV is still high even in the era of neurokinin-1 inhibitors. Failure of antiemetic control in the first cycle remains the main risk factor associated with the onset of symptoms. Therefore, every effort should be made to control nausea or CINV in the first cycle to prevent the symptoms prophylactically. More research is needed to evaluate other risk factors in the emergence of ANV and the impact of ANV on patients’ quality of life.
